# Improving event-based progression analysis in glaucomatous visual fields

**DOI:** 10.1038/s41598-021-95877-9

**Published:** 2021-08-11

**Authors:** Chiara Rui, Giovanni Montesano, David P. Crabb, Paolo Brusini, Balwantray C. Chauhan, Luca M. Rossetti, Paolo Fogagnolo, Jean-Marie Giraud, Jean-Rémi Fenolland, Francesco Oddone

**Affiliations:** 1 CenterVue SpA, Padua, Italy; 2 Optometry and Visual Science, City, University of London, Northampton Square, London, EC1V 0HB UK; 3 NIHR Biomedical Research Centre, Moorfields Eye Hospital NHS Foundation Trust and UCL Institute of Ophthalmology, London, UK; 4 Department of Ophthalmology, “Città di Udine” Health Center, Udine, Italy; 5 Department of Ophthalmology and Visual Sciences, Dalhousie University and Nova Scotia Health Authority, Halifax, Canada; 6 University of Milan e ASST Santi Paolo e Carlo, Milan, Italy; 7 Bégin Military Hospital, Glaucoma Center, Saint Mandé, France; 8 Val-de-Grâce Army Medical School, Paris, France; 9 Fondazione Bietti IRCCS, Rome, Italy

**Keywords:** Optic nerve diseases, Visual system, Visual system

## Abstract

Glaucoma is a progressive optic neuropathy with characteristic changes to the optic nerve head and the visual field (VF). Detecting progression of VF damage with Standard Automated Perimetry (SAP) is of paramount importance for clinical care. One common approach to detecting progression is to compare each new VF test to a baseline SAP test (*event analysis*). This comparison is made difficult by the test–retest variability of SAP, which increases with the level of VF damage, and the limited range of measurement, meaning that damage cannot be assessed below a certain level. We performed a prospective international multi-centre data collection of SAP data on 90 eyes from 90 people with glaucoma and different levels of VF damage over a short period of time (6 tests in 60 days). Data were collected using a fundus tracked perimeter (Compass, CenterVue). We used these data (minus the first test) to develop an improved event analysis that accounts for both the change in variability with damage and the lower bound on the measurement imposed by SAP. Using simulations, we show that our approach is more sensitive compared to previously developed methods, especially in the case of advanced glaucoma, while retaining similar specificity.

## Introduction

Glaucoma is a progressive optic neuropathy characterised by typical morphological changes to the optic nerve head (ONH) and the retina. Functional damage from glaucoma manifests as characteristic defects to the visual field (VF). Monitoring the progression of VF damage is of paramount importance for clinical management and is the main outcome of many glaucoma trials^1–8^. The VF is usually measured with Static Automated Perimetry (SAP). In SAP, circular stimuli of different intensities are projected at various predetermined locations on the retina. Efforts have been made to improve the reliability of the exam, for example with the use of fundus tracking technology to compensate for eye movements (Fundus tracked Automated Perimetry, FAP). The Compass fundus perimeter (CMP, CenterVue, Padova, Italy) is a FAP device that includes all conventional perimetric test patterns employed for testing glaucoma patients, such as the 24-2 of the Humphrey Field Analyzer (HFA, Carl Zeiss Meditec, Dublin, CA)^9^.

Despite advances in perimetric hardware and software, variability remains a major hurdle in assessing VF progression. The CMP has proven useful, compared to HFA, in reducing test–retest variability for global indices^9^, indicating a possible improvement from using FAP. However, measurements at individual locations retained the typical increased variability at lower sensitivities observed with SAP^9^. Although several aspects can contribute to long-term variability, the systematic relationship between sensitivity and variability is thought to be a consequence of the shallower psychometric function^10^ in locations with lower sensitivity. As a consequence, methods to assess pointwise progression of VF damage need to account for such differences in variability. Event-based analyses are common methods to assess point-wise progression. An example is the Guided Progression Analysis (GPA) algorithm implemented for the HFA which seeks to identify changes in sensitivity-related metrics that fall outside the expected 95% test–retest limits for a specific location, based on an estimate of baseline damage. Because this analysis is conducted pointwise, there is an increased chance of identifying locations with change exceeding the test–retest limits, even in stable patients, leading to False Positives (FP). Empirical rules have been implemented to curb this phenomenon, requiring a given number of locations to be outside the limits to classify progression^11–13^. However, these empirical rules do not account for the fact that variability at more damaged locations is so large that the 95% lower limits would fall outside the range testable by the VF device^14^. These locations would therefore not contribute to the False Positive Rate (FPR) and accounting for this characteristic could improve the detection of progression in more damaged VFs without compromising specificity.

For this study, we prospectively collected test–retest series with the CMP perimeter in a cohort of 90 stable glaucoma patients, stratified by VF damage. We used these data to evaluate a novel event-based approach that accounts for the number of locations in which progression can effectively be observed (*adaptive rule*) and compare this to a fixed decision rule and the empirical GPA decision thresholds. Progression was simulated with realistic rates of VF decay from an independent cohort of glaucoma patients.

## Results

### Sample description

Ninety eyes of 90 subjects (55 males, 35 females) were recruited. The sample was composed of 34 (37.8%) eyes with early glaucomatous loss, 28 (31.1%) eyes with moderate glaucomatous loss and 28 (31.1%) with advanced loss. The characteristics of the study sample are summarized in Table 1. All patients had 6 reliable VF tests but only 5 were used (the first practice test was discarded).

**Table 1:** Descriptive statistics of the test–retest sample.

	Early loss (N = 34)	Intermediate loss (N = 28)	Advanced loss (N = 28)
Sex (male:female)	19:16	17:11	19:8
Age (years)	65 [58.5, 72.5]	68 [64, 73.5]	66 [60.5, 75.5]
BCVA (logMAR)	0.0 [0.0, 0.02]	0.0 [0.0, 0.10]	0.0 [0.0, 0.10]
24-2 MD (dB)	− 3.5 [− 5.2, − 2.0]	− 8.2 [− 10.5, − 6.1]	− 13.6 [− 18.9, − 11.2]
24-2 PSD (dB)	7.2 [4.2, 8.9]	11.1 [9.8, 13.0]	12.4 [10.8, 13.2]

*IQR* interquartile range, *MD* mean deviation, *PSD* pattern standard deviation, *BCVA* best corrected visual acuity, *IOP* intra ocular pressure; Early glaucomatous loss: − 6 dB ≤ MD ≤ 0 dB; Intermediate glaucomatous loss: − 12 dB ≤ MD < − 6 dB; Advanced glaucomatous loss: MD < − 12 dB.

### Test–retest variability limits

**Figure 1:**
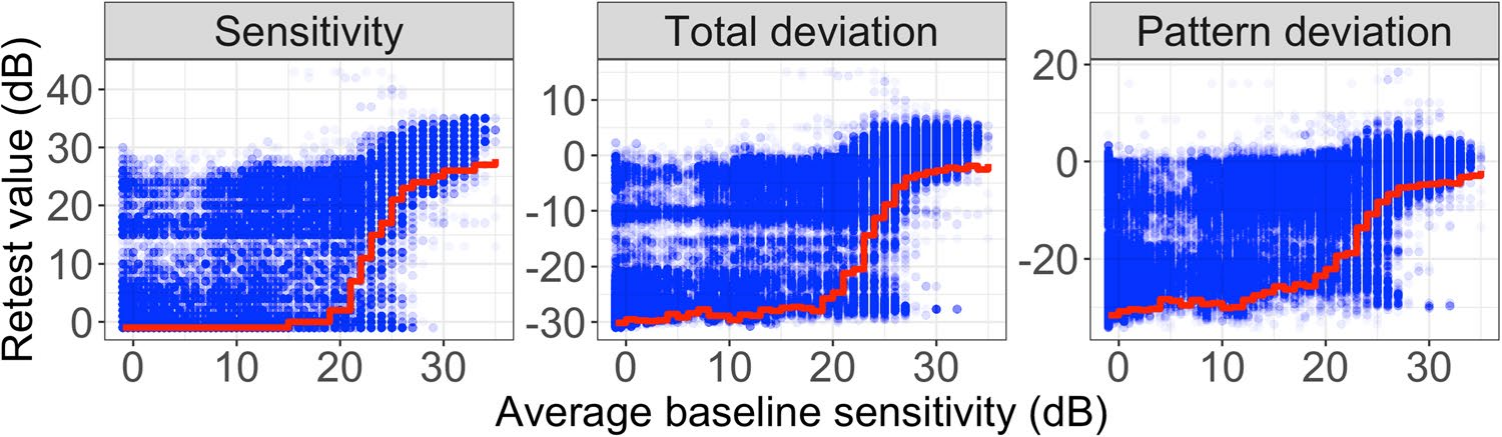
Test–retest distribution for sensitivity (left), Total deviation (middle) and Pattern deviation (right) values stratified by baseline sensitivities values (average of two tests). The red lines show the lower 5% limits derived for each baseline sensitivity values.

Figure 1 shows the test–retest distribution for the sensitivity, the Total Deviation (TD) and the Pattern Deviation (PD) values, stratified by baseline sensitivity. The test–retest distribution was obtained from all 10,800 permutations of the test–retest series. Baseline sensitivity was estimated by taking the average of the first two VFs of each permutated series. TD and PD are values derived from sensitivity. TD accounts for the effect of both normal ageing and eccentricity. PD also accounts for global changes in the VF, for example in the case of cataract, highlighting localized VF loss. As expected, the test–retest variability increased at lower sensitivities with all metrics. The test–retest limits were calculated as the 5% quantile of the test–retest distribution for each metric at each baseline sensitivity value. A floor effect is clearly visible for sensitivity values with an average baseline sensitivity < 15 dB, below which value the test–retest limit exceeds the dynamic range of the instrument. Details of the calculations are reported in the “Methods”. We performed an additional analysis (reported as supplementary material) to investigate factors associated with test–retest variability, measured as the absolute residuals for each location in each VF with respect to the average across the five repetitions, similarly to Choi et al.^15^. The average MD was the only significant predictor of increased variability (p = 0.014), but this significance was lost when stratifying by the average sensitivity of each location (p = 0.628). Changes in variability at different locations were also explored using Bland–Altman^16^ plots (supplementary material).

### Event analysis

Point-wise linear progression of VF damage was simulated at each location similarly to Wu et al.^17^. The pointwise slopes to simulate change over time were derived from the Longitudinal Glaucoma VFs (LGVFs) database from the Rotterdam Ophthalmic data repository^18,19^, a collection of 278 VF series from glaucoma patients tested with the Full Threshold (FT) algorithm with the HFA. Each eye in our sample was matched to one eye in the LGVFs dataset using the average sensitivity at baseline. Details of the simulation and matching methodology are reported in the “Methods” and supplementary material. The average pointwise progression rate for the matched sample was − 0.29 ± 1.24 dB/year. Series with simulated progression were used together with permutations of the stable test–retest series to determine the rate of detection of progression (Hit Rate, HR) and the FPR of the different event-based progression methods. A progression event was identified when sensitivity (or TD/PD value) fell below the test–retest limits for the baseline. We combined the results of different locations using different rules to account for the expected FPR, according to the number of locations considered: the *fixed rule* always considered all 52 locations; the *adaptive rule* excluded locations affected by the floor effect, where no progression could be assessed. We calculated the HR and FPR obtained by combining individual events (E1), and two or three consecutive events (E2 and E3 respectively). These were compared with an implementation of the GPA. Details are provided in the “Methods” and supplementary material.

**Figure 2:**
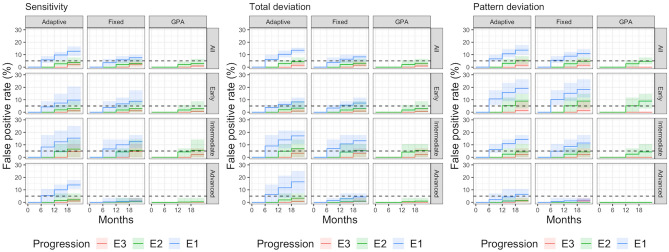
Cumulative percentage of FPR of the three progression analyses (Adaptive rule, Fixed rule and GPA) for sensitivity (left), Total Deviation (centre) and Pattern Deviation (right). Cumulative detection rates are reported for each follow-up visit (6, 12 and 18 months) and for each progression type (E1, E2 and E3). The horizontal dashed line indicates 5% FPR.

**Figure 3:**
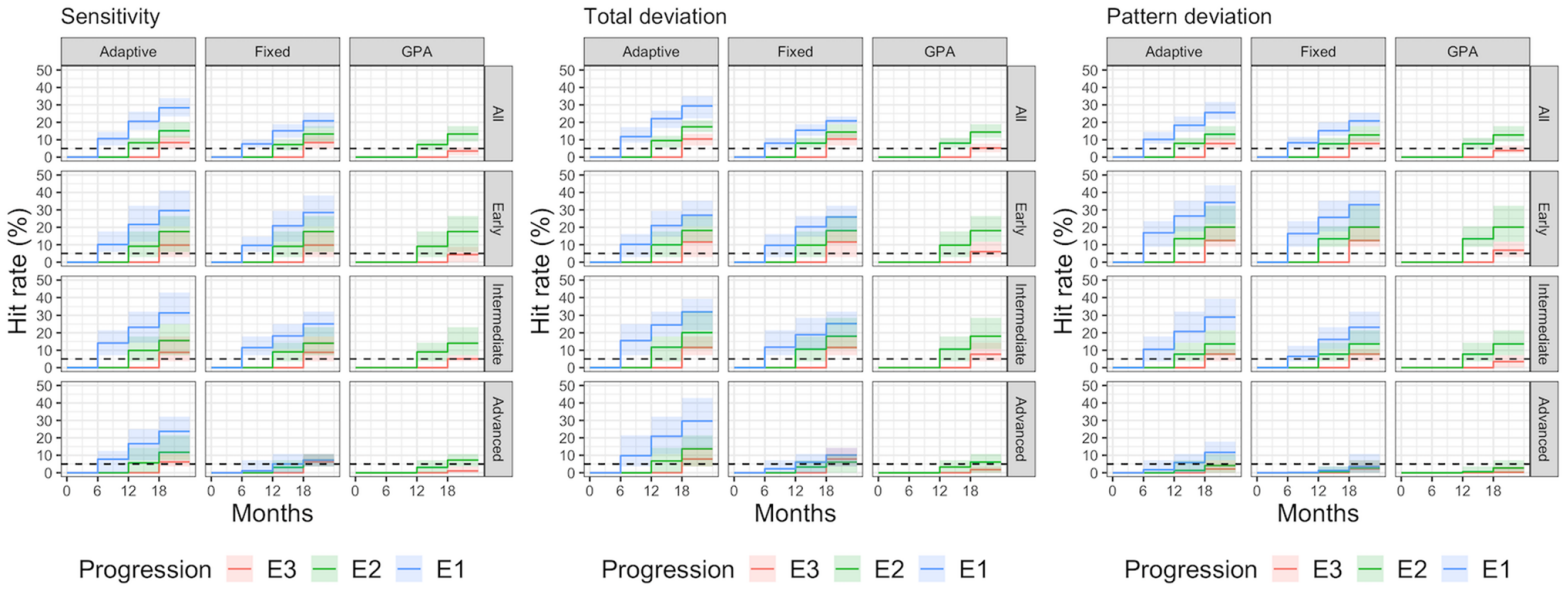
Cumulative percentage of HR of the three progression analyses (Adaptive rule, Fixed rule and GPA), for sensitivities (left), Total Deviation (centre) and Pattern Deviation (on the right). Cumulative detection rates are reported for each follow-up visit (6, 12 and 18 months) and for each progression type (E1, E2 and E3). The horizontal dashed line indicates 5% HR.

Figures 2 and 3 report the results of the event detection with the three algorithms for sensitivities, TD and PD as cumulative percentage of HR and FPR at each visit. The shaded bands represent the 95% quantiles around the central estimate from different realisations of the simulation (i.e., permutations of the template/field sequences), used to define the variability of the estimate.

The FPR for E1 at first follow-up (6 months) was in general close to the desired 5%. However, it was on average larger when the adaptive rule was applied to the sensitivities (8.2%) and TD maps (9%) in the group with intermediate damage and for the PD maps for eyes with early damage (10.2% for the fixed rule and 10.7% for the adaptive rule). As expected, the FPR was much lower than 5% for the advanced cases with the fixed rule (0.4% for the sensitivities, 1.4% for TD and 0.6% for PD). However, the 5% limit was outside the 95% variability range of the estimate only for the PD maps in subjects with early damage and in the advanced group for the fixed rule with any map. Beyond the first follow-up, the FPR for E1 steadily increased, as expected. The HR was generally higher for the adaptive rule. Importantly, the HR for the advanced cases when the adaptive rule was applied to the sensitivities (7.7%) and the TD values (9.8%) was close to that obtained in the overall sample (10.6% for sensitivities, 11.7% for TD). The HR was instead extremely reduced for the fixed rule (1.1% for sensitivities, 2.3% for TD) and the GPA (0% for sensitivities, 0% for TD).

The FPR for E2 at the second follow-up (12 months) was lower or very close to 5% in all cases and was very similar among all detection methods. The only meaningful difference, as expected, was observed for the advanced cases where the FPR was higher with the adaptive rule applied to the sensitivities (1.5% for the adaptive rule, and 0.2% for the fixed rule and GPA) and TD values (1.9% for the adaptive rule, and 0.5% for the fixed rule and GPA). Concordantly, the HR was also better for the adaptive rule in advanced cases (5.6% for the sensitivities and 6.7% for the TD), doubled compared to the fixed rule and the GPA (3% for the sensitivities and 3.3% for the TD for both methods).

For E3 at the last follow-up (18 months) the fixed and adaptive rules behaved very similarly across the whole spectrum of damage both in terms of FPR and HR, with marginally higher FPR and HR compared to GPA.

Tables with the full numerical results are provided in the supplementary material. The average (SD) number of locations available for progression was 45.7 (7.9) for patients with early glaucomatous loss, 37 (9) for patients with intermediate glaucomatous loss and 25.5 (11) for patients with advanced glaucomatous loss.

We performed an additional analysis (reported in supplementary material) to investigate progression with a point-wise trend analysis^20^, showing substantial equivalence between all metrics.

## Discussion

We compared different algorithms to detect event-based progression. We derived our data from test–retest series in 90 glaucoma patients, stratified by VF damage. Our simulations relied on a method that accounts for global fluctuations in sensitivity across different locations in the VF, estimated from the data^17^. This is particularly important to accurately estimate the performance of event-based analyses, which instead assume each location is independent.

The observed test–retest distribution for the Compass was very similar to that reported in a previous study with a smaller dataset^9^. The variability increased substantially for sensitivities below 25 dB, in accordance with previous literature^14,21,22^, reaching the floor for the lower 5% limits at 15 dB. Interestingly, such a value has often been reported as a lower bound for locations to be informative for progression detection^14,23^. Indeed, some newly implemented testing strategies have also considered not testing locations with sensitivities below 15 dB^24^ because of their limited usefulness. Our data confirm this view, showing that such locations are not informative for event-based progression. The test–retest variability was very similar between sensitivities, TD and PD. It is important to notice that, differently from the Swedish interactive thresholding algorithm (SITA) employed by the HFA, the CMP does not make use of any spatial smoothing or growth patterns in the testing strategy^9,25,26^. Testing each location independently could lead to an increase in test–retest variability at lower sensitivities despite fundus tracking as reported in our previous study^9^. However, locations below 15 dB have been shown to be scarcely informative for progression also with SITA strategies^14,27^, implying that such differences are unlikely to be relevant for practical applications. Differently from previous analyses and from the traditional GPA^21^, the expected lower 5% limits for TD and PD was modelled based on the average baseline sensitivity rather than the average TD or PD values. This reflects the idea that sensitivity, rather than TD or PD, is the main determinant of response variability^10^. This is supported by our supplemental analysis showing that no other factors were significant predictors of increased variability when accounting for sensitivity. It is important to note, however, that locations with the same sensitivity have been shown to have very different variability, such as in Chauhan et al.^28^. In their analysis, however, sensitivity was still a better parameter than TD to characterise systematic changes in variability. This supports our choice of baseline sensitivity as the best, albeit imperfect, parameter to model the expected test–retest variability.

When comparing the different methods of event-based analysis, we found that they performed similarly in the overall sample. The GPA for E3 offered a lower HR with minor improvement in the FPR compared to the other methods. Compared to previous reports on the performance of the GPA^21,29–31^, our analysis yielded similar values for the FPR and HR for “*likely progression*” detection (0% and 3.7% respectively in the overall sample with PD values). However, for “*probable progression*” the specificity was higher than previously reported. Artes et al.^30^ showed a cumulative FPR close to 5% at 2 and 3 cumulative tests, similar to our results. However, Wu et al.^29^ reported a FPR of approximately 8% at 2 and 3 cumulative tests. In our simulations, the FPR with the GPA “*probable progression*” never exceeded the 5% threshold, despite using a similar simulation methodology. Of course, several differences could have contributed to this discrepancy, including the different perimeter employed and the use of a tobit model to estimate and simulate progression instead of a logistic function and the different sample composition (see supplementary material). The use of the same dataset to define the test–retest limits and to test our detection strategies could have influenced the measured performance, but this effect was minimized by the use of a leave-one-out approach in the simulations. One novel aspect of our analysis is that we grouped locations based on the average baseline sensitivity to calculated the lower 5% limits for event detection. This is different from how variability for the PD values was handled for the GPA^21^, which was instead stratified according the average of baseline PD values. Our decision was taken in recognition of the fact that the major determinant of perimetric variability is the sensitivity^10^, independently of the general height or normal ageing.

Similarly to us, Wu et al. showed that the GPA had a smaller HR for more damaged fields, but did not investigate the corresponding change in specificity^29^. With our analysis, we show that such a reduction in the HR is connected to an unwanted increase in specificity at more advanced stages, derived from the static rules applied to detect progression. Indeed, such a feature was shared by our *fixed rule* detection. On the contrary, the *adaptive rule* maintained a more homogeneous FPR across the severity spectrum. This effect was absent for the E3 detection, which was unchanged between the *fixed* and *adaptive* rules, but resulted in important improvements for the advanced cases for the E1 and E2 detections. Interestingly, for early and advanced cases, the FPR for E1 detection was below the 5% threshold with the *fixed rule* and very close to 5% for the *adaptive rule* in the advanced group. The 5% limit was also always well within the 95% variability range of the FPR estimate form the simulations for all classes of damage. For the advanced class, this was accompanied by an important increase in HR. This has clinical relevance for the advanced cases, in which faster detection of VF deterioration would be desirable to prevent progression to significant visual impairment or blindness. Progression with E1 criteria had low specificity for patients with intermediate damage using sensitivity and TD values. This could be explained by the gradual shift towards more variable sensitivities. Importantly, except for the E1 criterion, both the HR and the FPR were virtually identical between the *fixed* and *adaptive rule* for intermediate and early patients. Moreover, both the *fixed* and *adaptive rule* generally outperformed the GPA, especially for the 3-Event detection, in terms of HR (higher) with minimal changes to the FPR, which remained < 5%. It must be noted that most of the methodology for the GPA was developed in the context of the Early Manifest Glaucoma Trial (EMGT)^32^ which was meant to detect progression in patients with early glaucomatous damage^12,32^. It is therefore unsurprising that such a methodology would perform poorly in intermediate and advanced cases, proving often too conservative for clinical applications.

The performance of detection with PD was worse for all detection criteria compared to that with sensitivities and TD. The detrimental effect of using PD was particularly evident for patients with early damage, in which specificity was generally lower compared to sensitivities and TD, and in advanced cases, in which the HR was reduced. This is in agreement with previous reports, showing that PD based metrics might hide progression in glaucoma patients^14,31^. One aspect to consider is that our dataset did not contain patients with visually significant cataract. However, the reported effect of lens opacity on the VF has been variable, ranging from significant^33–36^ to negligible^37,38^, with a measured effect on global indices, such as the MD, of approximately 1 dB. Therefore, the role of metrics meant to account for generalised VF depression, such as the PD, remains unclear in the context of determining glaucoma progression, also in consideration of the potential masking effect of such indices^31^ and their limited reliability in advanced glaucoma damage.

One limitation of our analysis is that the length of the test–retest series did not allow for permutations/simulations of more than 5 visual fields per patient. Further differences between the methods might arise in longer series. However, this study was meant to test the performance of event-based analyses for early detection of progression, where they would be more clinically relevant. Moreover, longitudinal VF series collected with the Compass in glaucoma patients are not yet available and simulation of progression had to rely on data collected with an HFA^19,39^. However, we based our simulations on noise estimates from the observed test–retest distribution, in an attempt to reliably mimic what would really be observed in a real-life use of the device.

In conclusion, our proposed *adaptive rule* has the potential to be more useful in the clinical practice than current implementations of the event analysis, such as the GPA, especially in patients with advanced damage. Sensitivities and TD values offered similar performance, but TD has the potential for wider application by accounting for normal ageing. Fundus-tracked perimetry might also better meet the assumption, in event-based analyses, that the same retinal locations are being tested at all follow-ups. However, from our previous report^9^, this feature did not translate to a clear reduction in pointwise test–retest variability over HFA. Therefore, the effective clinical improvement brought by fundus tracking in detecting progression needs to be determined with longitudinal VF series on progressing glaucoma patients and comparing data collected with and without the use of fundus tracking technology.

## Methods

### Data collection for test–retest variability

Data were collected as part of a longitudinal open-label international multicentre study. Five sites took part in the study: IRCCS Fondazione “G. B. Bietti”, Rome, Italy; Polyclinic Città di Udine, Udine, Italy; Ophthalmology Service and Glaucoma Center HIA Begin. Paris, France; Eye Care Centre, Nova Scotia Health, Halifax, Canada; and ASST Santi Paolo e Carlo, Milan, Italy. Recruitment started on August 30, 2018 and ended on December 20, 2019. We enrolled 90 consecutive patients with glaucoma, testing one eye per subject. The study eye was randomly selected if both eyes met the inclusion criteria. Eyes were stratified into three groups according to disease severity, using the 24-2 Mean Deviation (MD) of the most recent reliable HFA test: (1) early glaucomatous loss (− 6 dB ≤ MD ≤ 0 dB); (2) moderate glaucomatous loss (− 12 dB ≤ MD < − 6 dB); (3) advanced glaucomatous loss (MD < − 12 dB).

Inclusion criteria were as follows: (1) Age between 18 and 80 years; (2) Best corrected visual acuity logMAR ≤ + 0.1 (≥ 8/10 decimal); (3) Spherical refraction between − 12D and + 15D (limits of autofocus in the CMP); (4) Astigmatism between − 2D and + 2D; (5) Clinically determined glaucomatous optic nerve damage; (6) Abnormal optical coherence tomography (OCT) scan of the retinal nerve fibre layer (RNFL) and optic nerve head (ONH) in at least one sector, with reference to the normative values specific of each device used; (7) At least 5 available reliable VF tests within the last 5 years; and (8) repeatable VF defects in the last 2 reliable VF tests, defined as: (i) MD or pattern standard deviation (PSD) outside 95% confidence limits (p < 0.05); (ii) Glaucoma Hemifield Test (GHT) outside normal limits; and (iii) A cluster of at least 3 points with p < 0.05 in the PSD map, one of each with p < 0.01 affecting the same hemifield, however, the cluster could not be contiguous with the blind spot and could not cross the horizontal midline^40^.

Exclusion criteria were as follows: (1) Any ocular surgery except uncomplicated cataract surgery and/or glaucoma surgery performed less than 6 months prior to data collection; (2) Any ocular pathology that could affect the VF other than glaucoma; (3) Use of any drug that could hinder the perimetric examination or its results; and (4) Angle-closure glaucoma.

Eligible patients were identified from the clinical registry of the glaucoma clinics in each recruiting centre. All patients gave written informed consent to participate in the study. Ethics Committee approval was obtained for each centre from the local ethical committee (Approval from the coordinating centre: Comitato Etico Centrale IRCCS Lazio, Sezione IRCCS I.F.O., June, 26, 2018, ref: N. 68 /18/FB). This study adhered to the tenets of the Declaration of Helsinki.

At the first visit, each subject underwent the following examinations in both eyes (besides the VF test): autorefraction, best corrected visual acuity, clinical assessment of the optic disc with slit lamp/ophthalmoscopy/fundus picture, OCT of the RNFL and ONH if a recent scan (< 3 months from data collection) was not available, Goldman applanation tonometry and gonioscopy.

### Visual field testing protocol

**Figure 4:**
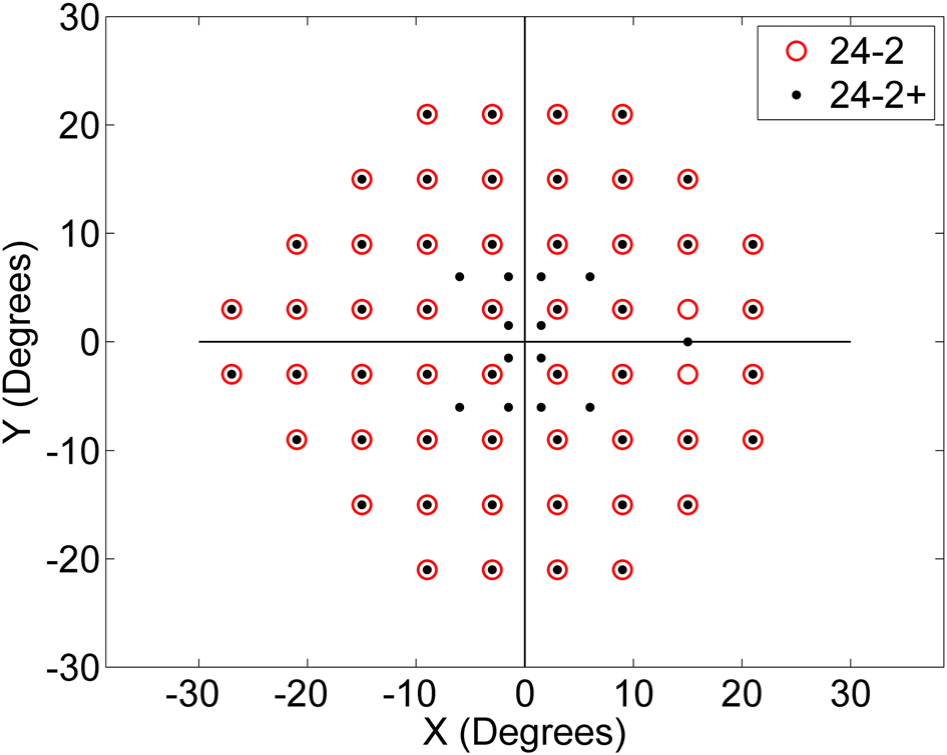
Comparison between standard 24-2 and 24-2+ grid.

VF examinations were performed with the CMP using a ZEST (Zippy Estimation through Sequential Testing)^25,41^ strategy and a 24-2+ grid. The 24-2+ grid has the same 52 locations of the standard 24-2 grid (excluding the two blind spot locations) with 12 additional points in the macular region (Fig. 4). If the patient was eligible, a first test (Practice) and 5 additional retests were performed within a period of about 1 month (average 31 days, range [16–44] days) with a mean interval of 6 days (range [1–15] days) between tests. All tests were considered reliable if the rate of false positive errors was ≤ 18%, the rate of false negative errors was ≤ 30% and the average pupil diameter during each test was > 2.8 mm (the threshold to ensure reliable imaging and tracking with the CMP). The tests were performed in follow-up mode, so that the same retinal locations were tested in each examination.

The Practice test was not included in the analysis and served to familiarise the patient with the testing procedure.

### Simulation of progressing fields

Simulations were performed using the method proposed by Wu et al.^17^ to obtain longitudinal visual field series. The details are reported in the supplementary material. Briefly, the method uses noise templates to simulate variability in the VFs while maintaining correlations across locations within the same test. This is an important factor in VF variability, sometimes referred to as the Global Visit Effect^42^, since the fluctuations in sensitivity at different visits are not independent across different locations. The method also accounts for the larger variability at lower sensitivity values and derives the noise distributions from the empirical test–retest data to specifically simulate the noise observed in CMP from this data collection. The pointwise slopes for sensitivity change over time were derived from the LGVFs database^18,19^, a collection of 278 VF series from glaucoma patients tested with the Full Threshold (FT) algorithm with the HFA. Both FT and the ZEST procedure of CMP derive sensitivity at test locations independently and without reference to neighbouring points. The pointwise slopes were calculated through tobit regression^39^ in R (R Foundation for statistical computing, Vienna, Austria) using the AER package^43^ to account for the floor effect at lower sensitivities (see supplementary material).

From the LGVFs, we selected 90 subjects matched with the participants of our test–retest dataset according to their baseline Mean Sensitivity (MS). For the CMP test–retest series, the MS was calculated as the mean of the 52 sensitivity values from the 24-2 locations, each estimated as the average value across all the VFs in the test–retest series from the same eye. For the LGVFs, the MS was the mean of the 52 values predicted by the tobit regression at baseline (first visit). The pointwise slopes from the matched LGVFs subjects were used to simulate progression in the eyes from our test–retest dataset. For our simulations, each VF in the original test–retest series was transformed into a noise template (see supplementary material). We simulated all possible combinations of noise templates (i.e. VFs) for each eye, for a total of 10,800 simulated VFs series (120 per eye). This allowed us to retain the VF fluctuations actually observed in each real test–retest series. Essentially, the simulated progressing VF series replicated the permutations of the test–retest series, with the additional effect of the simulated decay in sensitivity. We simulated two baseline visits at 0 and 6 months, and then three more VF at 12, 18 and 24 months (total of 5 VFs, corresponding to the 5 noise templates per eye).

### Event analysis

An event analysis method was developed to detect significant progression events. Based on the method proposed by Heijl et al.^21^, we used the average of two VFs in the test–retest series to define the baseline sensitivity for each location in each eye. The lower 5% limits for the test–retest distribution were calculated for each baseline sensitivity and used to detect significant progression *events*. The same calculations were repeated using the TD and the PD values, as provided by the CMP using an internal normative database^9^. Of note, when calculating the 5% limits, TD and PD values were grouped according to their average baseline sensitivity instead of the average TD and PD baseline values. This choice was preferred since sensitivity is known to be the main determinant of response variability in perimetry^10^. The limits were calculated for each eye by excluding all data from that same eye, to avoid inflating the performance (leave-one-out). Additional details are provided in the supplementary material.

We compared different criteria to define a VF series as progressed. Progression could be identified by 1 event (E1) or two (E2) or three (E3) consecutive events at the same location. A set of *fixed decision rules* were used to combine these events at multiple locations:

- E1 progression: one event is observed in at least 6 locations
- E2 progression: two consecutive events are observed in at least 3 locations
- E3 progression: three consecutive events are observed in at least 2 locations

**Figure 5:**
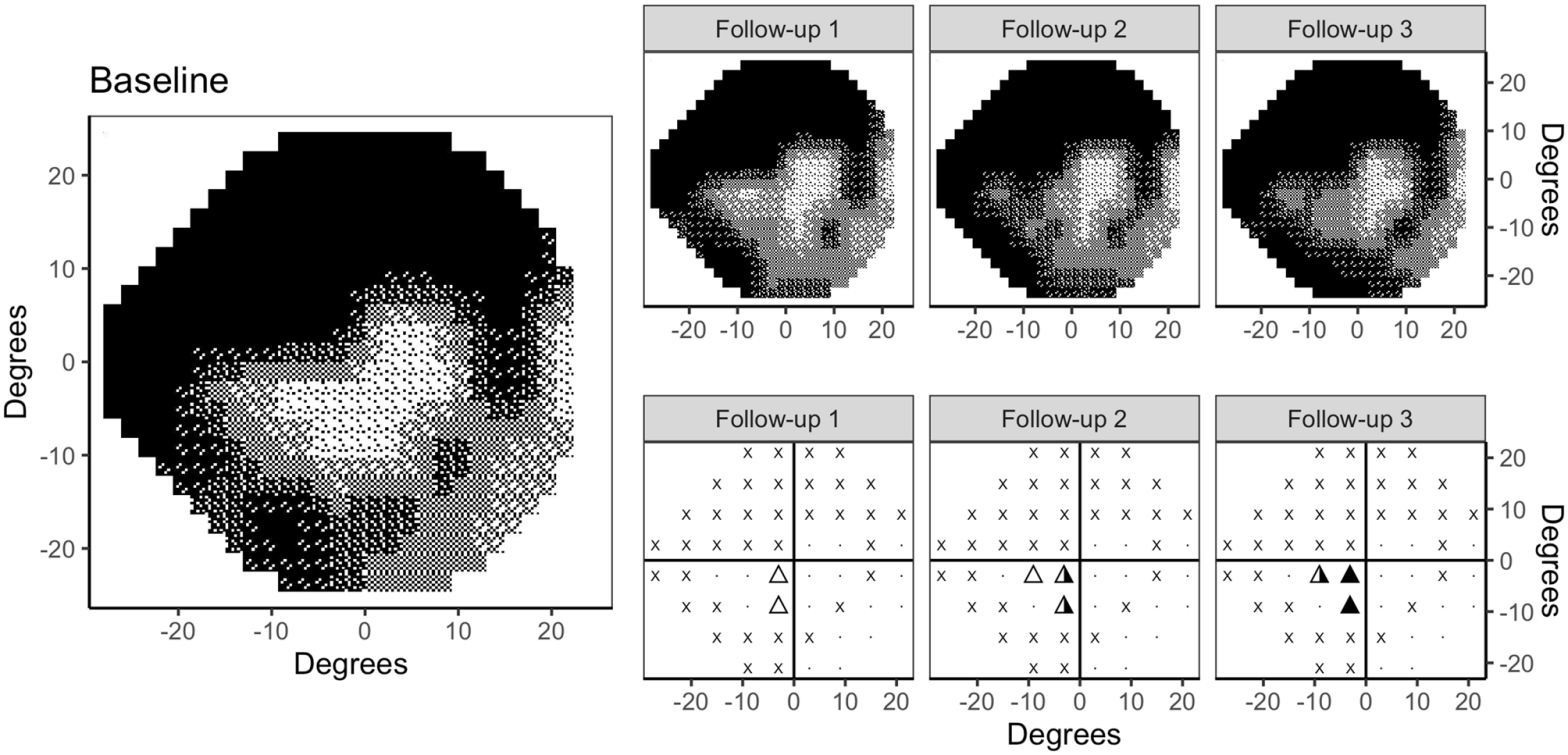
Example of event analysis for an eye with advanced damage with simulated progression. The grayscale plots shows the visual field at baseline (average of the first two tests) and at the three follow-ups (6, 12 and 18 months). For each follow-up test the event analysis is reported at the bottom. Empty, half-filled and filled triangles indicate, respectively, one, two and three consecutive events at the same location. An event is defined as significant deterioration beyond the 5th percentile of sensitivity test–retest limits. The crosses indicate locations where detection of an event is not possible. GPA detected an E2 progression (“possible progression”) at 18 months; the *Fixed rule* detected an E3 progression at 18 months; the *Adaptive rule* detected an E2 progression at 12 months and an E3 progression at 18 months (first detection 6 months earlier).

Similar to the GPA, the *fixed rule* is meant to account for the rate of expected false positives in the detection of the events, considering each location (N = 52) as a separate statistical test with a 5% probability of false positive error. The probability of detecting at least one false *event* was assumed to follow a binomial distribution (see supplementary material for details). However, the *fixed rule* does not account for locations where detecting an event is impossible (locations with sensitivity at Baseline below 15 dB), because the lowest 5% quantile of the test–retest distribution coincides with the lower measurement limit (0 dB). These locations do not contribute to the overall false positive rate and are more frequent in eyes with advanced damage, because lower sensitivities have larger test–retest variability (Fig. 5). We therefore proposed a modification of this rule (the second algorithm in our comparison, called *adaptive rule*) where the minimum number of locations with an observed event required to detect progression is adjusted according to the effective number of locations in which progression can be measured (potentially less than 52). Details are reported in the supplementary material. Finally, we compared our results with the empirical rules reported for GPA^11–13^: no VF progression is based on E1; E2 and E3 progression is assessed in both cases if two (“possible progression”) or three (“likely progression”) consecutive events respectively are observed in at least 3 locations. This differs from our fixed E3 criterion, where only 2 locations are required.

We applied these algorithms to all permutations of test–retest series, assumed stable, to evaluate the FPR and to all simulated progressing series to measure the rate of detection (Hit Rate, HR). For each series, the first two tests were used as baselines for the event analysis, over a total of 5 tests. Therefore, E1 progression could be detected for test 3 to 5, E2 for tests 4 and 5 and E3 only for test 5. We considered 5% as the ideal benchmark for FPR for clinical use. The variability of our estimates is reported using the 5–95% quantiles of the results obtained from different realisations of our simulations (i.e., each permutation of the VF series).

## Supplementary Information

Supplementary Information.
