# Supplementary material for: Improving event-based progression analysis in glaucomatous visual fields
Source: Sci Rep. 2021 Aug 11;11:16353. doi: 10.1038/s41598-021-95877-9 (PMC8357820; doi:10.1038/s41598-021-95877-9)
Supplement: Supplementary file 1 — Supplementary Information. [file 41598_2021_95877_MOESM1_ESM.docx]

Supplementary material

# Simulation approach

Simulations of progressing visual field (VF) series were based on the methodology proposed by Wu et al.^1^ For each patient (N=90 VFs series collected with Compass) and each location, an estimate of the true threshold is calculated as the rounded average of the five values obtained from the test-retest series, assumed stable. For each location in each VF, the residuals are then calculated as the difference from the estimated true threshold. These residuals are then grouped according to their rounded estimated true threshold to create empirical distribution functions (eCDF) of perimetric noise for each sensitivity level. An example of the noise eCDF for a sensitivity threshold of 30 dB is reported in Supplementary Figure 1. This allowed us to model the specific perimetric noise for the Compass.


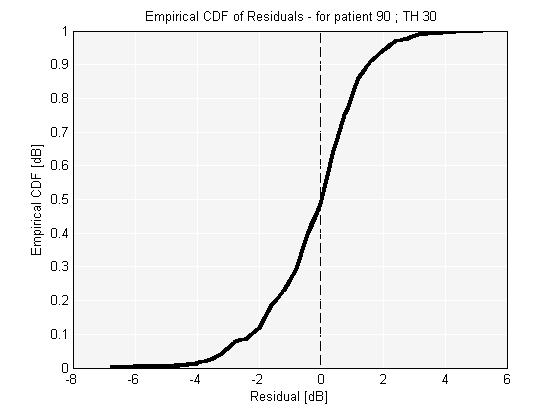


**Supplementary Figure 1** Example of an empirical Cumulative Distribution Function (eCDF) used to map the residuals (horizontal axis) to the corresponding standardized score (vertical axis) and vice-versa. This eCDF represents the noise distribution for 30 dB sensitivity.

These eCDFs were used to transform each observed sensitivity value into a score normalized between 0 and 1. In the example in Supplementary Figure 1, this is obtained by mapping the values of the residuals along the horizontal axis onto the vertical axis using the eCDF specific for each sensitivity. For example, in the figure, a residual of +1 dB would be mapped to 0.75, whereas a residual on 0 dB would be mapped to 0.50. The eCDFs become shallower at lower sensitivities, reflecting the change in variability. These normalized scores can then replace the actual sensitivity values for each VF to create a noise template that is independent of the specific threshold values. A noise template can be derived from each real VF test (5 per subject). These noise templates can then be applied, via inverse mapping, to simulated fields to introduce noise. For the inverse process, the eCDF corresponding to the simulated threshold is selected and the normalized score is back-transformed into a residual value that can then be added to the threshold. This process is performed for all the locations in the simulated VF. This method preserves the noise correlations between locations within the same VF, since the noise templates are applied as a whole to the simulated field. Progression can be simulated by degrading the sensitivity at each location using a linear mode (see below). An example of the noise templates derived from a real test-retest series is reported in Supplementary Figure 2.


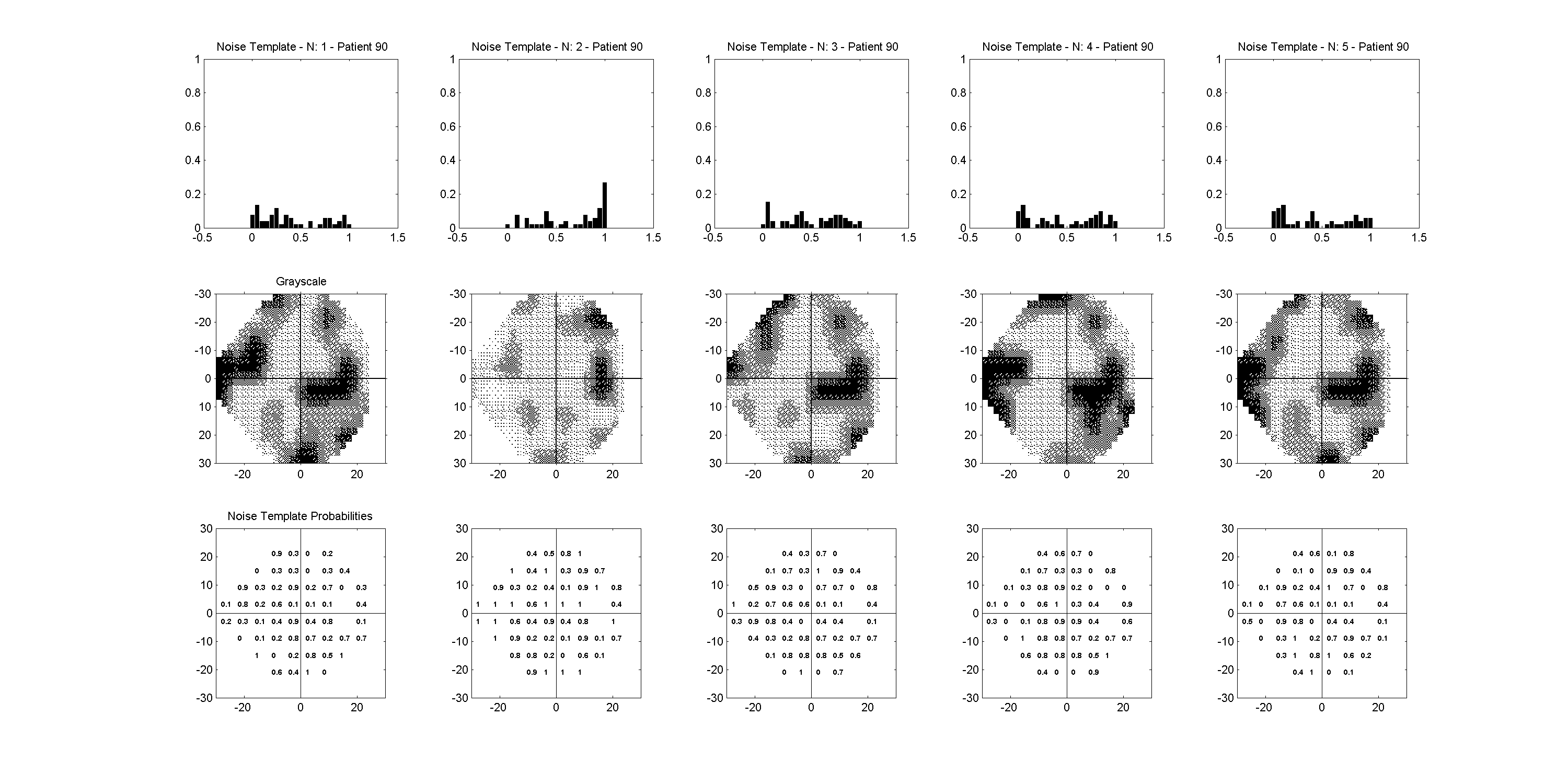


**Supplementary Figure 2** Visual fields from a stable test-retest series (middle row) and the corresponding normalized scores for the residuals (histograms, top row). The noise templates are represented in the bottom row. Notice how the second test shows a global fluctuation towards higher thresholds, correctly modelled by the noise template, whose values are globally shifted towards 1.

To simulate realistic VF deterioration, we estimated the point-wise rate of progression from the Longitudinal Glaucomatous VFs data set (LGVFs) from the Rotterdam Ophthalmic data repository ^2,3^. The LGVFs is a dataset of glaucomatous eyes tested with a Humphrey Field Analyzer (HFA) device using a full-threshold algorithm. The dataset was composed of 278 eyes (139 patients) followed up for a mean period of 9 years, with on average more than 17 fields per eye (17.5 ± 2.3 VFs). For all eyes, we calculated the pointwise rate of progression using a censored linear regression (tobit function from the package AER ^4^), which accounts for the fact that sensitivities < 0 dB are not observed (Supplementary Figure 3). This ensured that the calculated rates of progression for fields with advanced baseline damage were not affected by the measurement floor^5^. These pointwise rates of progression were then used to degrade the VF of our stable test-retest cohort. All 90 eyes in our test-retest dataset were matched with a corresponding eye in the LGVFs based on the Mean Sensitivity (MS) at baseline. The rates of progression from the matched eyes were used to simulate progression. The matching procedure was meant to account for the fact that eyes with worse VF damage at baseline might progress at a faster rate. The MS at baseline was calculated as the average of the intercepts at baseline for the progressing eyes in the Rotterdam dataset and as the average of the “true” estimated point-wise threshold for the stable eyes in the test-retest dataset.


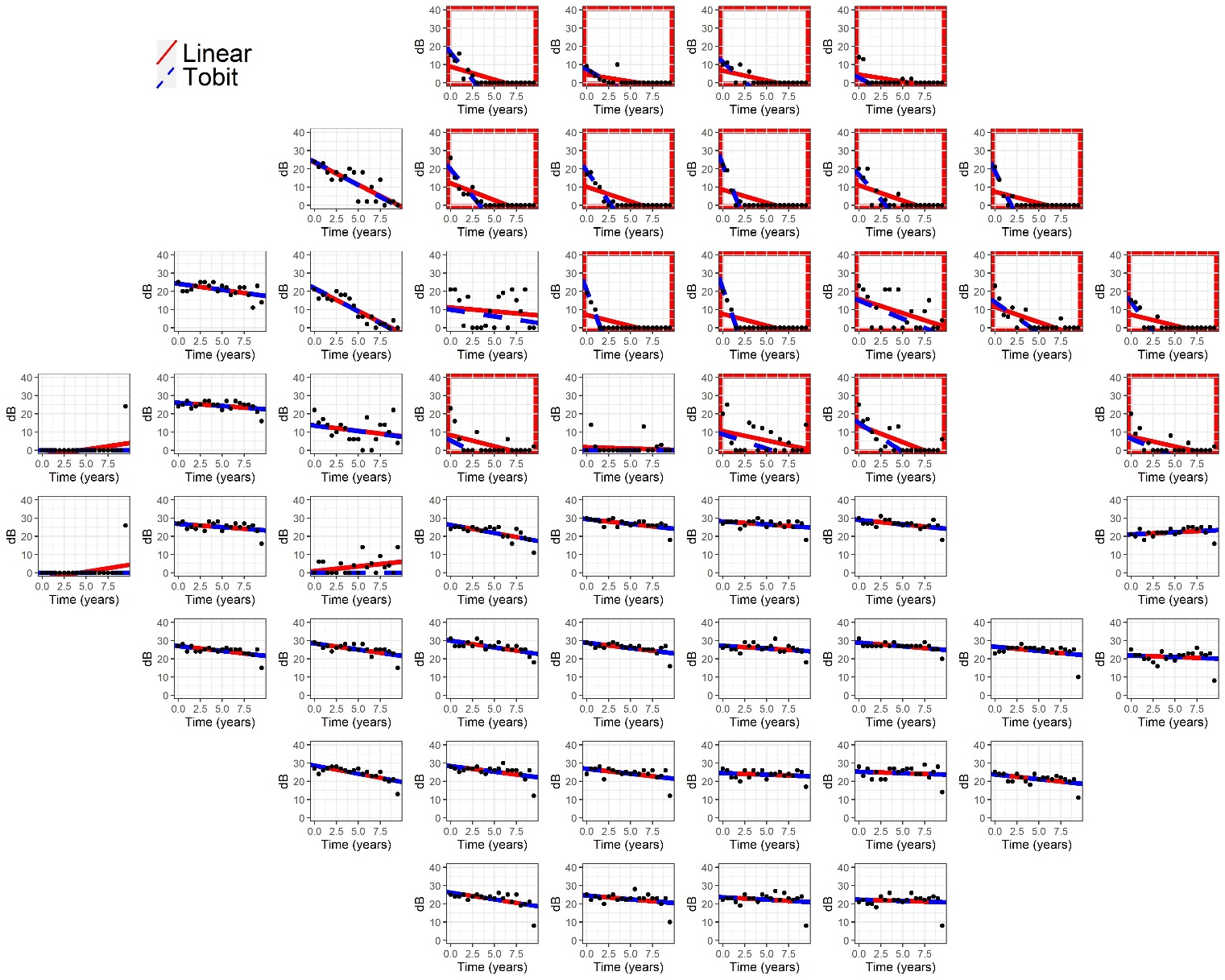


**Supplementary Figure 3** Comparison between classical point-wise linear regression and censored regression for one series in the Rotterdam dataset. The red square highlights a location where the progression rate calculated with usual linear regression is affected by the measurement floor, due to trailing zeros. In contrast, censored regression correctly measures the rapid deterioration by accounting for censored values < 0 dB.

Realistic variability was injected in the simulations using the noise templates derived from each stable series of the test-retest dataset, as explained above. Progression in each eye was simulated using only the 5 noise templates derived from the test-retest series of that same eye. All permutations of noise templates were employed. In this sense, the progressing series exactly mirrored the permutations of the stable test-retest series except for the simulated VF deterioration. VFs were simulated at 0, 6, 12, 18 and 24 months.

# Event analysis

An event analysis method was developed to detect significant progression events. Following Hejl et al.^6^, we used the average of two VFs in the test-retest series to define the baseline sensitivity for each location in each eye. We rounded the baseline raw threshold values to the closest integer and calculated, for each sensitivity value, the lowest 5% quantile using all the threshold values from all remaining VF tests. This value was then used as a lower bound to identify significant changes in sensitivity from the baseline at any follow-up (*event*). The limits were calculated using all possible permutations of the series (N = 120 per eye), so that all possible VF pairs were used as baselines. The same calculations were repeated using the Total Deviation (TD) and the Pattern Deviation (PD) values, as provided by the CMP using an internal normative database^7^. Differently from previous studies, the 5% limits for TD and PD values were calculated by grouping these values according to their average raw sensitivity of the two baseline VFs rather than using the average of TD and PD baseline values. This choice was preferred since raw sensitivity is known to be the main determinant of response variability in perimetry^8^. The lower 5% limits used for our analysis were computed using a leave-one-out approach, so that the subject being evaluated was not included in the calculation of the limits.

## Detection of progression

As explained in the manuscript, we adopted a set of *fixed decision rules* to detect progression based on one, two or three consecutive events at the same location (E1, E2 and E3 progression respectively):

- E1 progression: one event is observed in at least 6 locations
- E2 progression: two consecutive events are observed in at least 3 locations
- E3 progression: three consecutive events are observed in at least 2 locations

The *fixed rule* is meant to account for the rate of expected false positives in the detection of the events, considering each location (N = 52) as a separate statistical test with a 5% probability of false positive error. For example, the formula used to decide the minimum number of progressed locations for the E1 criterion is based on the inverse cumulative distribution function (CDF) of a binomial distribution with an outcome probability of 5% for 52 independent trials. The inverse binomial is then evaluated at p = 1 – 0.05 to obtain the minimum number of progressed locations needed. However, such a rule does not account for correlations among different locations within the same test^1,9^, which are instead modelled in our simulations. This reduces the expected specificity of detection especially for the E2 and E3 criteria. We accounted for this by evaluating the inverse CDF at more stringent p, defined as p = 1 – 0.05^(N of consecutive events)^. This leaves the decision rule unaltered for E1, but becomes p = 1 – 0.0025 for E2 and p = 1 – 0.000125 for E3. The number of locations required with the *adaptive rule* was instead calculated by changing the number of trials in the inverse CDF formula (i.e. less than or equal to 52).

# Cumulative False positives and Hit-rate

The tables below report the cumulative hit-rate (HR) and false positive (FP) rate for each metric and the three detections methods (note that “Adaptive” has been shortened “Adapt.”).

|  |  |  | **Sensitivity** | | | | | | | | | | | | | | |
| --- | --- | --- | --- | --- | --- | --- | --- | --- | --- | --- | --- | --- | --- | --- | --- | --- | --- |
|  |  |  | **All** | | | | **Early damage** | | | | **Intermediate damage** | | | | **Advanced damage** | | |
|  | **Visit** |  | **Adap.** | **Fixed** | **GPA** | **Adap.** | | **Fixed** | **GPA** | **Adap.** | | **Fixed** | **GPA** | **Adap.** | | **Fixed** | **GPA** |
| **E1** | **3** | **FP** | 5.8 | 3.6 | - | 4.2 | | 3.7 | - | 8.2 | | 6.7 | - | 5.4 | | 0.4 | - |
|  | **4** |  | 9.7 | 5.9 | - | 7.4 | | 6.7 | - | 12.4 | | 10.0 | - | 10.0 | | 0.7 | - |
|  | **5** |  | 12.8 | 7.6 | - | 9.7 | | 8.8 | - | 15.4 | | 12.5 | - | 13.9 | | 1.1 | - |
|  | **3** | **HR** | 10.6 | 7.6 | - | 10.1 | | 9.7 | - | 14.1 | | 11.5 | - | 7.7 | | 1.1 | - |
|  | **4** |  | 20.6 | 15.1 | - | 21.6 | | 20.9 | - | 23.2 | | 18.2 | - | 16.6 | | 5.0 | - |
|  | **5** |  | 28.3 | 20.8 | - | 29.6 | | 28.5 | - | 31.4 | | 25.1 | - | 23.7 | | 7.2 | - |
| **E2** | **4** | **FP** | 2.7 | 2.1 | 2.1 | 1.9 | | 1.8 | 1.8 | 4.8 | | 4.3 | 4.3 | 1.5 | | 0.2 | 0.2 |
|  | **5** |  | 3.7 | 2.9 | 2.9 | 2.8 | | 2.8 | 2.8 | 6.5 | | 5.6 | 5.6 | 2.0 | | 0.4 | 0.4 |
|  | **4** | **HR** | 8.3 | 7.2 | 7.2 | 9.1 | | 9.1 | 9.1 | 9.9 | | 9.0 | 9.0 | 5.6 | | 3.0 | 3.0 |
|  | **5** |  | 15.1 | 13.2 | 13.2 | 17.5 | | 17.5 | 17.5 | 15.6 | | 14.0 | 14.0 | 11.7 | | 7.2 | 7.2 |
| **E3** | **5** | **FP** | 2.2 | 2.2 | 0.9 | 1.2 | | 1.2 | 0.6 | 4.6 | | 4.6 | 2.1 | 1.1 | | 1.1 | 0.0 |
|  | **5** | **HR** | 8.4 | 8.4 | 3.5 | 9.8 | | 9.8 | 4.3 | 8.8 | | 8.8 | 5.0 | 6.2 | | 6.2 | 1.0 |

**Supplementary Table 1.** False positive (FP) and Hit rate (HR) for each event-based method for different disease severity group applied to sensitivity. Adapt. = Adaptive; GPA = Guided Progression Analysis; E = Event.

|  |  |  | **Total deviation** | | | | | | | | | | | | | | |
| --- | --- | --- | --- | --- | --- | --- | --- | --- | --- | --- | --- | --- | --- | --- | --- | --- | --- |
|  |  |  | **All** | | | | **Early damage** | | | | **Intermediate damage** | | | | **Advanced damage** | | |
|  | **Visit** |  | **Adap.** | **Fixed** | **GPA** | **Adap.** | | **Fixed** | **GPA** | **Adap.** | | **Fixed** | **GPA** | **Adap.** | | **Fixed** | **GPA** |
| **E1** | **3** | **FP** | 6.2 | 3.9 | - | 3.7 | | 3.3 | - | 9.0 | | 6.9 | - | 6.3 | | 1.4 | - |
|  | **4** |  | 10.3 | 6.4 | - | 6.3 | | 5.7 | - | 13.9 | | 10.7 | - | 11.7 | | 2.9 | - |
|  | **5** |  | 13.6 | 8.2 | - | 8.2 | | 7.4 | - | 17.1 | | 13.2 | - | 16.4 | | 4.3 | - |
|  | **3** | **HR** | 11.7 | 8.0 | - | 10.2 | | 9.7 | - | 15.5 | | 11.7 | - | 9.8 | | 2.3 | - |
|  | **4** |  | 22.1 | 15.4 | - | 21 | | 20.3 | - | 24.5 | | 18.9 | - | 20.9 | | 6.0 | - |
|  | **5** |  | 29.4 | 20.8 | - | 27 | | 25.9 | - | 32 | | 25.2 | - | 29.6 | | 10.1 | - |
| **E2** | **4** | **FP** | 2.9 | 2.1 | 2.1 | 2.0 | | 1.8 | 1.8 | 4.9 | | 4.2 | 4.2 | 1.9 | | 0.5 | 0.5 |
|  | **5** |  | 4.4 | 3.0 | 3.0 | 3.2 | | 2.9 | 2.9 | 6.8 | | 5.5 | 5.5 | 3.3 | | 1.0 | 1.0 |
|  | **4** | **HR** | 9.5 | 8.0 | 8.0 | 10 | | 9.8 | 9.8 | 11.7 | | 10.7 | 10.7 | 6.7 | | 3.3 | 3.3 |
|  | **5** |  | 17.4 | 14.3 | 14.3 | 18.2 | | 18.1 | 18.1 | 20.1 | | 18.1 | 18.1 | 13.7 | | 6.1 | 6.1 |
| **E3** | **5** | **FP** | 1.6 | 1.6 | 0.9 | 0.9 | | 0.9 | 0.6 | 3.2 | | 3.2 | 2.1 | 0.7 | | 0.7 | 0.0 |
|  | **5** | **HR** | 10.4 | 10.4 | 5.2 | 11.6 | | 11.6 | 6.0 | 11.6 | | 11.6 | 7.7 | 7.9 | | 7.9 | 1.7 |

**Supplementary Table 2.** False positive (FP) and Hit rate (HR) for each event-based method for different disease severity group applied to total deviation. Adapt. = Adaptive; GPA = Guided Progression Analysis; E = Event.

|  |  |  | **Pattern deviation** | | | | | | | | | | | | | | |
| --- | --- | --- | --- | --- | --- | --- | --- | --- | --- | --- | --- | --- | --- | --- | --- | --- | --- |
|  |  |  | **All** | | | | **Early damage** | | | | **Intermediate damage** | | | | **Advanced damage** | | |
|  | **Visit** |  | **Adap.** | **Fixed** | **GPA** | **Adap.** | | **Fixed** | **GPA** | **Adap.** | | **Fixed** | **GPA** | **Adap.** | | **Fixed** | **GPA** |
| **E1** | **3** | **FP** | 6.6 | 5.6 | - | 10.7 | | 10.2 | - | 6.2 | | 4.9 | - | 2.1 | | 0.6 | - |
|  | **4** |  | 10.7 | 8.8 | - | 15.9 | | 15.2 | - | 11.0 | | 8.7 | - | 4.3 | | 1.2 | - |
|  | **5** |  | 13.6 | 11.0 | - | 19.1 | | 18.2 | - | 14.3 | | 11.4 | - | 6.5 | | 1.8 | - |
|  | **3** | **HR** | 10.2 | 8.3 | - | 16.9 | | 16.4 | - | 10.5 | | 6.5 | - | 1.7 | | 0.1 | - |
|  | **4** |  | 18.3 | 15.2 | - | 26.5 | | 25.7 | - | 20.7 | | 16.2 | - | 5.8 | | 1.3 | - |
|  | **5** |  | 25.6 | 20.8 | - | 34.3 | | 33.0 | - | 29.0 | | 23.2 | - | 11.6 | | 3.6 | - |
| **E2** | **4** | **FP** | 3.1 | 2.7 | 2.7 | 5.2 | | 5.1 | 5.1 | 2.5 | | 2.5 | 2.5 | 1.1 | | 0.0 | 0.0 |
|  | **5** |  | 5.1 | 4.7 | 4.7 | 8.9 | | 8.9 | 8.9 | 4.3 | | 4.3 | 4.3 | 1.4 | | 0.0 | 0.0 |
|  | **4** | **HR** | 7.9 | 7.6 | 7.6 | 13.5 | | 13.4 | 13.4 | 7.7 | | 7.7 | 7.7 | 1.4 | | 0.5 | 0.5 |
|  | **5** |  | 13.1 | 12.7 | 12.7 | 20.1 | | 20.1 | 20.1 | 13.6 | | 13.6 | 13.6 | 4.1 | | 2.7 | 2.7 |
| **E3** | **5** | **FP** | 1.6 | 1.6 | 0.0 | 1.5 | | 1.5 | 0.0 | 2.1 | | 2.1 | 0.0 | 1.1 | | 1.1 | 0.0 |
|  | **5** | **HR** | 7.8 | 7.8 | 3.7 | 12.5 | | 12.5 | 6.9 | 7.8 | | 7.8 | 3.5 | 2.1 | | 2.1 | 0.2 |

**Supplementary Table 3.** False positive (FP) and Hit rate (HR) for each event-based method for different disease severity group applied to pattern deviation. Adapt. = Adaptive; GPA = Guided Progression Analysis; E = Event.

# Point-wise trend analysis

We assessed the detection of progression using the Permutation Analyses of Pointwise Linear Regression (PoPLR)^10^ as implemented on the visualFields package for R^11^, using sensitivity, Total deviation (TD) and Pattern deviation (PD) metrics. HR-Specificity curves were plotted (**supplementary figure 4**) for different cut-offs on the global p-values obtained from PoPLR. The circular targets indicate the conventional cut-off at p = 0.05. Of note, the better performance with sensitivity is likely due to a combined effect of glaucoma progression and ageing, which is not present in the stable series but part of the rates used to simulate progression.

**
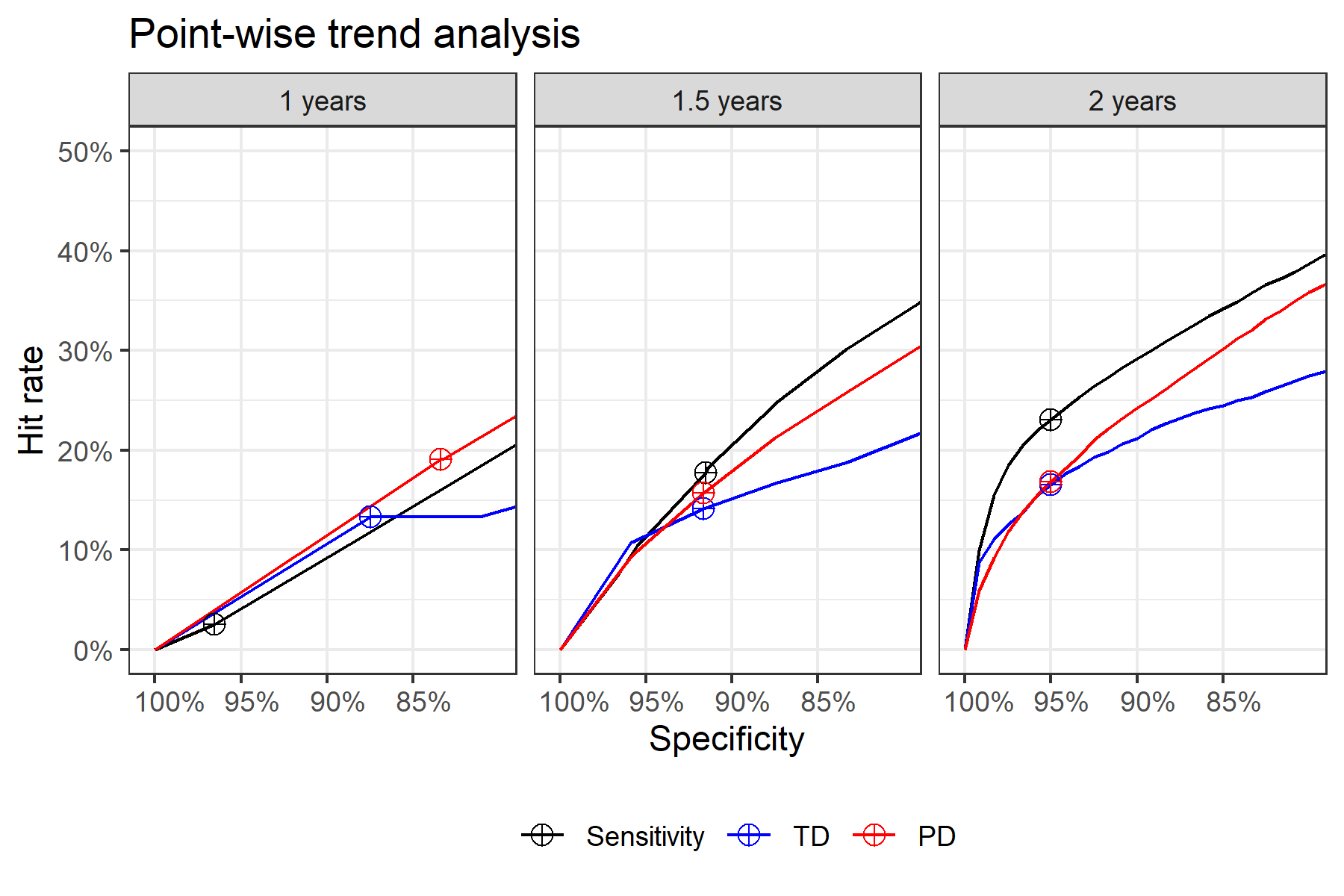
**

**Supplementary Figure 4.** Hit-rate – specificity plots for sensitivity, total deviation (TD) and pattern deviation (PD) for at three 1, 1.5 and 2 years of follow up.

# Factors affecting variability

Variability was assessed by modelling the absolute residuals, calculated as the difference between the sensitivity values and their average across the five test repeats. A multivariate linear mixed model **(Model 1**, with random effects to account for multiple observations from the same eye) was used to investigate the effect of age, Mean Deviation, Pattern Standard Deviation, False Positive and False Negative errors and log_10_(Bivariate Contour Ellipse Area), a metric of fixation stability^12^. All these parameters were quantified by taking the average across different test repeats. A second model (**Model 2**) also included the rounded average sensitivity as a categorical predictor. In the first model, the Mean Deviation was the only significant predictor (p = 0.0142). However, this significance was lost in the second model, where the average sensitivity was the only significant determinant of variability (overall p < 0.001 ). The table below reports the effects and p-values of the predictors for the two models. For the second model, the effect of each sensitivity value (modelled as a level of a categorical factor) has been omitted for simplicity.

|  | **Effect on absolute residuals** | | | |
| --- | --- | --- | --- | --- |
|  | **Model 1** | | **Model 2** | |
|  | **Coefficient** | **P** | **Coefficient** | **P** |
| **Mean deviation (dB)** | -0.22 [-0.4, -0.05] | **0.0142** | 0.04 [-0.13, 0.22] | 0.6279 |
| **Pattern standard deviation (dB)** | 0.29 [-0.03, 0.61] | 0.0838 | 0.26 [-0.07, 0.58] | 0.1239 |
| **Age (years)** | 0.01 [-0.08, 0.1] | 0.8869 | 0.01 [-0.08, 0.1] | 0.8557 |
| **Mean FP (%)** | 0.05 [-0.33, 0.43] | 0.7992 | 0.04 [-0.34, 0.42] | 0.8309 |
| **Mean FN (%)** | -0.15 [-0.33, 0.03] | 0.1094 | -0.13 [-0.31, 0.05] | 0.1701 |
| **log_10_(Mean BCEA)** | -0.67 [-2.52, 1.19] | 0.4833 | -0.47 [-2.34, 1.4] | 0.6203 |

**Supplementary Table 4.** Effect of different predictors of variability. The effects are presented as estimate [95% Confidence Intervals]. Model 1 shows the effect without accounting for the average sensitivity of each location. Model 2 stratifies different locations by sensitivity using a discrete fixed effect. FP = False positive; FN = False negative; BCEA = Bivariate Contour Ellipse Area.

## Variability per location of the visual field

Bland-Altman plots^13^ for different locations of the 24-2 grid. The labels report the 95% Limits of Agreement (LoA), calculated as the 2.5% and 97.5% quantiles of all test-retest pair differences.

**
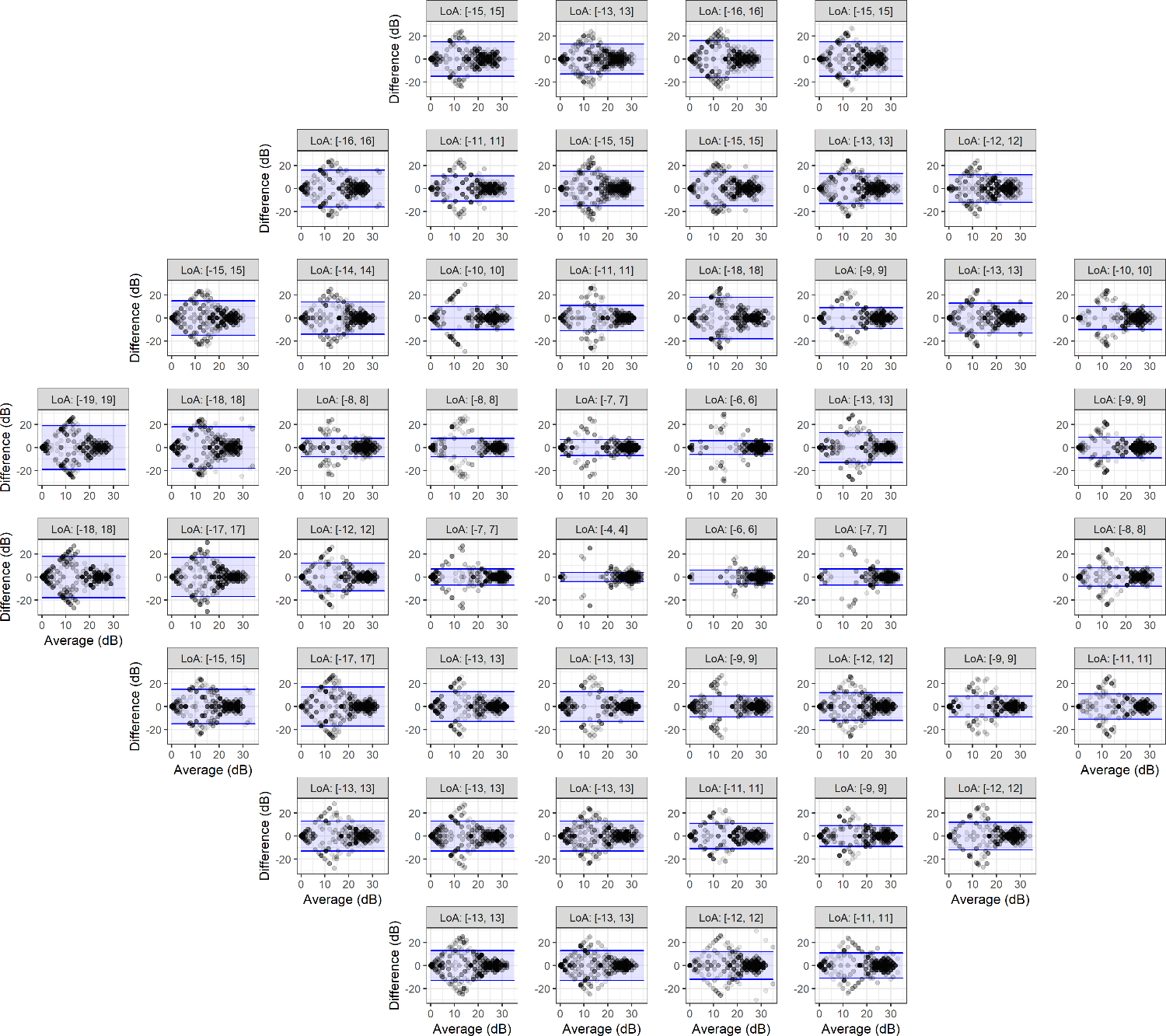
**

**Supplementary Figure 5.** Each panel represents the Bland-Altman plot for all different test-retest pairs in the sample (in all possible pairing orders). The shaded band represents the 95% Limits of Agreement (LoA), also reported in the panel title.

# References

1 Wu, Z. & Medeiros, F. A. Development of a Visual Field Simulation Model of Longitudinal Point-Wise Sensitivity Changes From a Clinical Glaucoma Cohort. *Transl Vis Sci Technol* **7**, 22, doi:10.1167/tvst.7.3.22 (2018).

2 Erler, N. S. *et al.* Optimizing structure-function relationship by maximizing correspondence between glaucomatous visual fields and mathematical retinal nerve fiber models. *Invest Ophthalmol Vis Sci* **55**, 2350-2357, doi:10.1167/iovs.13-12492 (2014).

3 Bryan, S. R., Vermeer, K. A., Eilers, P. H., Lemij, H. G. & Lesaffre, E. M. Robust and censored modeling and prediction of progression in glaucomatous visual fields. *Invest Ophthalmol Vis Sci* **54**, 6694-6700, doi:10.1167/iovs.12-11185 (2013).

4 Kleiber, C. & Zeileis, A. Applied Econometrics with R. *Springer-Verlag* (2008).

5 Russell, R. A. & Crabb, D. P. On alternative methods for measuring visual field decay: Tobit linear regression. *Invest Ophthalmol Vis Sci* **52**, 9539-9540, doi:10.1167/iovs.11-8948 (2011).

6 Heijl, A., Lindgren, A. & Lindgren, G. Test-retest variability in glaucomatous visual fields. *Am J Ophthalmol* **108**, 130-135, doi:10.1016/0002-9394(89)90006-8 (1989).

7 Montesano, G. *et al.* A Comparison between the Compass Fundus Perimeter and the Humphrey Field Analyzer. *Ophthalmology* **126**, 242-251, doi:10.1016/j.ophtha.2018.08.010 (2019).

8 Henson, D. B., Chaudry, S., Artes, P. H., Faragher, E. B. & Ansons, A. Response variability in the visual field: comparison of optic neuritis, glaucoma, ocular hypertension, and normal eyes. *Invest Ophthalmol Vis Sci* **41**, 417-421 (2000).

9 Bryan, S. R., Eilers, P. H., Lesaffre, E. M., Lemij, H. G. & Vermeer, K. A. Global Visit Effects in Point-Wise Longitudinal Modeling of Glaucomatous Visual Fields. *Invest Ophthalmol Vis Sci* **56**, 4283-4289, doi:10.1167/iovs.15-16691 (2015).

10 O'Leary, N., Chauhan, B. C. & Artes, P. H. Visual field progression in glaucoma: estimating the overall significance of deterioration with permutation analyses of pointwise linear regression (PoPLR). *Invest Ophthalmol Vis Sci* **53**, 6776-6784, doi:10.1167/iovs.12-10049 (2012).

11 Marin-Franch, I. & Swanson, W. H. The visualFields package: a tool for analysis and visualization of visual fields. *J Vis* **13**, doi:10.1167/13.4.10 (2013).

12 Crossland, M. D., Sims, M., Galbraith, R. F. & Rubin, G. S. Evaluation of a new quantitative technique to assess the number and extent of preferred retinal loci in macular disease. *Vision Res* **44**, 1537-1546, doi:10.1016/j.visres.2004.01.006 (2004).

13 Bland, J. M. & Altman, D. G. Statistical methods for assessing agreement between two methods of clinical measurement. *Lancet* **1**, 307-310 (1986).
